# Scientific Items

**Published:** 1877-09-20

**Authors:** 


### Scientific Items

FEELING THE PULSE BY TELEGRAPH.—Some months ago Dr. Upham, of Salem, Mass., in order to explain to his audidience the variations of pulse in certain diseases, caused the lecture room to be placed in telegraphic communication with the city hospital of Boston, distant fifteen miles, and by means of special apparatus the various pulse beats were exhibited by a vibrating ray of magnesium light upon the wall. These experiments have lately been repeated at Paris with success.—*New Rem.*

OXYGEN IN THE SUN.—Dr. Draper has published a paper, with illustrated diagrams, in *American Journal of Science and Arts*, in which he shows that oxygen, in large proportions, exists in the sun, and probably nitrogen also. It is regarded as lending further and powerful confirmation to the nebular hypothesis. The combination of appliances by which the learned observer arrived at his conclusions evinced great ingenuity, laborious application and scientific skill, and it is believed opens the way to other important and perhaps startling discoveries in regard to the constitution of the heavenly bodies.

WAITEHEAD TORPEDO—Is a cigar-shaped machine about twelve feet long. In the head is a charge of dynamite, which explodes with terrible power whenever it strikes an obstacle. The machine is shot from a tube, and moves in a straight line through the water at any depth desired. If it fails to strike the object, it rises to the surface without exploding.

LONGEVITY OF MAN.—Conclusions, drawn from insurance tables and other statistical observations, indicate a decided advance in the longevity of the human race. Two centuries ago in London the death rate was 50 per 1,000, and the average of life twenty years. The average of life in America is now about orty-two years.

PETRIFACTION.—Amonst the varied and interesting collection in the Geological Department of Georgia is a petrified turtle, of large size, and many other rare specimens of petrifaction.

CALCIUM—*A Compound.*—In the photographic spectrum of the star (*a Lyræ*) one of the lines of calcium is wanting, indicating that calcium is a compound, and not an elementary body, as heretofore regarded.

JETTIES.—The jetties at New Orleans are so far advanced that a depth of 20½ feet of water has been secured, admitting vessels of much larger tonnage than ever before.

POPULATION OF THE GLOBE.—It is estimated that the present population of the earth is 1,428,800,000.

